# Supplementary material for: Experimental study of precursory features of CO2 blasting-induced coal rock fracture based on grayscale and texture analysis
Source: PLoS One. 2024 Feb 9;19(2):e0297753. doi: 10.1371/journal.pone.0297753 (PMC10857609; doi:10.1371/journal.pone.0297753)
Supplement: S1 File — (DOCX) [file pone.0297753.s001.docx]

The following is the MATLAB code for extracting texture feature parameters in RGB strain image.

clc

clear

rgb=imread('1.jpg');

Gray = double(0.3*rgb(:,:,1)+0.59*rgb(:,:,2)+0.11*rgb(:,:,3));

%--------------------------------------------------------------------------

%To reduce the amount of computation, the Gray level of the original image is compressed and quantized to 64 levels

%--------------------------------------------------------------------------

for i = 1:443

for j = 1:748

for n = 1:256/64

if (n-1)*64<=Gray(i,j)&&Gray(i,j)<=(n-1)*64+15

Gray(i,j) = n-1;

end

end

end

end

[L,num]=bwlabel(Gray);

%--------------------------------------------------------------------------

%Four co-occurrence matrices P are calculated, taking the distance as 1 and the angles as 0,45,90,135 respectively

%--------------------------------------------------------------------------

P = zeros(64,64,4);

for m = 1:64

for n = 1:64

for i = 1:1435

for j = 1:485

if j<748&&Gray(i,j)==m-1&&Gray(i,j+1)==n-1

P(m,n,1) = P(m,n,1)+1;

P(n,m,1) = P(m,n,1);

end

if i>1&&j<485&&Gray(i,j)==m-1&&Gray(i-1,j+1)==n-1

P(m,n,2) = P(m,n,2)+1;

P(n,m,2) = P(m,n,2);

end

if i<1435&&Gray(i,j)==m-1&&Gray(i+1,j)==n-1

P(m,n,3) = P(m,n,3)+1;

P(n,m,3) = P(m,n,3);

end

if i<1435&&j<485&&Gray(i,j)==m-1&&Gray(i+1,j+1)==n-1

P(m,n,4) = P(m,n,4)+1;

P(n,m,4) = P(m,n,4);

end

end

end

if m==n

P(m,n,:) = P(m,n,:)*2;

end

end

end

%%---------------------------------------------------------

%Normalization of the co-occurrence matrix

%%---------------------------------------------------------

for n = 1:4

P(:,:,n) = P(:,:,n)/sum(sum(P(:,:,n)));

end

%----------------------------------------------------------------------

%Four texture parameters, ASM, CON, COR and ENT, were calculated for the co-occurrence matrix

%--------------------------------------------------------------------------

H = zeros(1,4);

I = H;

Ux = H; Uy = H;

deltaX= H; deltaY = H;

C =H;

for n = 1:4

E(n) = sum(sum(P(:,:,n).^2));

for i = 1:64

for j = 1:64

if P(i,j,n)~=0

H(n) = -P(i,j,n)*log(P(i,j,n))+H(n);

end

I(n) = (i-j)^2*P(i,j,n)+I(n);

Ux(n) = i*P(i,j,n)+Ux(n);

Uy(n) = j*P(i,j,n)+Uy(n);

end

end

end

for n = 1:4

for i = 1:64

for j = 1:64

deltaX(n) = (i-Ux(n))^2*P(i,j,n)+deltaX(n);

deltaY(n) = (j-Uy(n))^2*P(i,j,n)+deltaY(n);

C(n) = i*j*P(i,j,n)+C(n);

end

end

C(n) = (C(n)-Ux(n)*Uy(n))/deltaX(n)/deltaY(n);

end
