# Supplementary material for: Experimental study of precursory features of CO2 blasting-induced coal rock fracture based on grayscale and texture analysis
Source: PLoS One. 2024 Feb 9;19(2):e0297753. doi: 10.1371/journal.pone.0297753 (PMC10857609; doi:10.1371/journal.pone.0297753)
Supplement: S2 File — (DOCX) [file pone.0297753.s002.docx]

This is the MTALAB code that converts the RGB strain image to grayscale image and plots the normalized gray histogram.

clc

clear

close all

srcImage=imread('1.png');

% RGB strain image conversion grayscale image

grayImage=rgb2gray(srcImage);

a=imcomplement(grayImage);

imwrite(a,'D:\desktop\g3.jpg');

info=imfinfo('D:\desktop\g3.jpg');

% Plot normalized gray histogram

[M,N]=size(grayImage);

[counts,x]=imhist(grayImage,64);

counts=counts/M/N;

set(stem(x,counts),'marker','none')
