# Supplementary material for: Experimental study of precursory features of CO2 blasting-induced coal rock fracture based on grayscale and texture analysis
Source: PLoS One. 2024 Feb 9;19(2):e0297753. doi: 10.1371/journal.pone.0297753 (PMC10857609; doi:10.1371/journal.pone.0297753)
Supplement: Attachment — Submitted filename: Commets-PONE-D-23-21600.pdf [file pone.0297753.s004.pdf]

Comments on PONE-D-23-21600

The paper entitled “Experimental Study of Precursory Features of CO_2 Blasting-induced Coal Rock Fracture based on Grayscale and Texture Analysis” discusses the study on enhancing gas extraction from coal seams using CO_2 blasting. The study involves laboratory experiments, numerical simulations, and the analysis of strain field grayscale and texture feature parameters over time. The research establishes a CO_2 blasting-induced coal rock fracture pressure model, a gas pressure distribution model, and examines the evolution of strain field characteristics during the blasting process. From my evaluation, it is an interesting topic and can inspire the investigation of this area. Overall, this paper is well prepared and well written. My estimation for this paper is Minor Revision. However, to accept this manuscript for publication, the following comments should be addressed first.

1. There are some grammar, spelling, and sentence structure errors. The authors should carefully double-check the paper.
2. This paper lacks comparative data to benchmark the effectiveness of CO_2 blasting against other gas extraction methods or industry standards.
3. This paper presents models for fracture pressure and gas pressure distribution, the validation of these models against field data or similar studies is not discussed. Without validation, the accuracy and reliability of these models could be questionable.
4. The conclusion suggests considering enclosed box environments and adsorption characteristics for future experiments. However, the document could have provided

more detailed insights into potential research directions to address the shortcomings and limitations of the current study.

5. This paper lacks a comprehensive introduction that establishes the context and significance of the research. Readers may find it challenging to grasp the importance of the topic without a clear background explanation.
