# Supplementary material for: Experimental study of precursory features of CO2 blasting-induced coal rock fracture based on grayscale and texture analysis
Source: PLoS One. 2024 Feb 9;19(2):e0297753. doi: 10.1371/journal.pone.0297753 (PMC10857609; doi:10.1371/journal.pone.0297753)
Supplement: Attachment — Submitted filename: Review comments.docx [file pone.0297753.s005.docx]

Review comments

This article uses gray and texture analysis methods, combined with *CO*2 blasting, and through theoretical analysis and laboratory experiments, it shows the process of *CO*2 blasting-induced coal rock fracture, and obtains the precursory characteristics of cracks from initiation to expansion. The scientific nature of the work is good. I think this research is meaningful, but this article needs to be minor revisions so that it can be published.

***1.*** *Compared with the original manuscript, the grammatical problems of the revised manuscript have been greatly improved, but there are still some small problems that need to be modified.*

***2.*** *Previous studies on CO2 induced fracturing have done a lot of work. It is suggested that the author should evaluate objectively in the citation, and highlight the characteristics of the research and the difference from the existing research.*

***3.*** *It is recommended to upload a clearer version of Figure 7 for publication.*

***4.*** *How many experiments were performed? Please add description in experiment section.*

***5.*** *Were any saturation performed before any experiment?*

***6.*** *Conclusion The description is too complicated, and it is suggested to reorganize the language.*

***7.*** *It is suggested that the last paragraph of the conclusion be re-summarized into a chapter and placed before the conclusion, which will be clearer. Just like this:*

Discussion

“The main strain field grayscale and texture features parameters can quantitatively characterize the coal rock cracking and propagation during the CO_2_ blasting process and determine the times of coal rock cracking and propagation. The goal is to improve blasting permeability and gas extraction efficiency while avoiding environmental and safety issues caused by CO_2_ misuse. The study provides a theoretical basis for enhancing gas extraction from coal seams. However, due to the limitations of simulation experiment conditions, a limitation of this study is that it solely employed uniaxial loading. The findings can be extrapolated to scenarios involving the fracturing of shallowly buried coal and rock, as well as coal rock containing numerous vertical faults, residual coal pillars, inclined coal seam slopes, and analogous rock-like materials without confining pressure. Nonetheless, for a more extensive range of applications, it becomes imperative to account for confining pressure within a closed-box environment, alongside the ramifications of CO_2_ adsorption by coal. Additionally, the coal seam's adsorption characteristics, mechanical properties, and relevant conditions such as gas concentration should be considered to provide better theoretical guidance for CO_2_ blasting permeability enhancement technology.”
