# Supplementary material for: Experimental study of precursory features of CO2 blasting-induced coal rock fracture based on grayscale and texture analysis
Source: PLoS One. 2024 Feb 9;19(2):e0297753. doi: 10.1371/journal.pone.0297753 (PMC10857609; doi:10.1371/journal.pone.0297753)
Supplement: Attachment — Submitted filename: Response to Reviewers.docx [file pone.0297753.s006.docx]

Manuscript Title: Experimental study of precursory features of CO_2_ blasting-induced coal rock fracture based on grayscale and texture analysis

Manuscript ID: PONE-D-23-21600

**Reviewer #7**

Manuscript entitled ‘Experimental study of precursory features of CO_2_ blasting-induced coal rock fracture based on grayscale and texture analysis’ discussed the possibility of applying GLCM method to identify the precursor characteristics of coal rock cracking and crack propagation. The study suffers some fundamental flaws that hinder the acceptance of this paper, detailed comments and suggestions are attached below for the authors’ reference.

Thank you very much for reviewing the manuscript and giving us the opportunity to revise and improve its quality. We have revised the article in terms of technology and writing. Please review it.

Issue 1. In the introduction section, too many Chinese references, and most literature are also from Chinese authors, it is suggested that the authors shall expand their reading scope and exclude some Chinese references.

*Response*:

Thank you for your diligent review and valuable suggestions. We have replaced some references.

Indeed, a substantial portion of the references cited in my paper are derived from Chinese literature or authored by researchers based in China.

China stands out globally as one of the countries most severely affected by coal and gas outburst disasters. The gas geological reserves within 2000 m of burial depth are estimated to be approximately 36.81 trillion cubic meters, comparable to the onshore conventional natural gas reserves of 38 trillion cubic meters. However, due to the low permeability of coal seams (2-3 orders of magnitude lower than in the United States), extracting gas from these coal seams presents significant challenges, posing a serious threat to mine safety and production.

The focus of my paper is on investigating a method to enhance coal seam permeability, particularly applicable to regions with low coal seam permeability, such as many coalfields in China. So most of the references are from Chinese authors. These references from Chinese authors are from highly influential journals.

Issue 2. L102 in P10, deep and tight coal seams? what does it mean? Coal seams with gas that is difficult to be extracted?

*Response*:

The issue you pointed out is true. We have made changes in the manuscript. We have changed “deep and tight coal seams” to “deep and low permeability coal seams”.

With the gradual increase of coal mining depth in China, the situation of complex geological structure, high gas pressure and low seam permeability in deep coal mines is becoming more serious. The difficulty of coalmine disaster prevention and control represented by coal and gas outburst is gradually increasing, which seriously restricts the safe and efficient coal mining in China. To improve the permeability of coal seam is the key to eliminate the coal and gas outburst risk and improve the efficiency of coalbed methane extraction. CO_2_ blasting is an effective method to increase coal seams permeability and improve gas drainage efficiency.

Issue 3. Fig.1 is an existing theory, reference shall be added here.

*Response*:

Fig.1 is a mathematical model drawn based on the experimental content to facilitate readers to understand the symbols and meanings appearing in the following formulas. The rock tensile fracture criterion formula involved in Fig.1 is an existing formula, and we have added references in the manuscript.

Issue 4. The method of obtaining Fig. 2 shall be explained, current layout of the curves is wield.

*Response*:

We have added the parameters of the blasting tube in **Sample preparation** section.

Based on these parameters and in conjunction with equations 12-15, the curve in Fig. 2 can be calculated.

Issue 5. The experimental design in this study had serious errors, the setup was too simply, only one specimen was prepared for each group, it would be better if the authors add some explanations here why only one specimen was selected from each group.

*Response*:

We conducted at least 5 experiments under each experimental condition. And typical results were selected for analysis. A description was added.

Figure 1. Some specimens (with and without white paint sprayed on the surface)

Issue 6. L219 in P17, why only the upper half of the specimen was selected for analysis?

*Response*:

Due to the connection of the blasting tube in front of the specimen, the pipeline covered the lower part of the specimen, leading to the loss of strain cloud map data. Therefore, we opted to analyze the upper portion of the specimen.

Figure 2. The specimen with the blasting tube

Issue 7. L221 in P17, how did the authors control the pressure and set the value as 1.5 MPa?

*Response*:

The CO_2_ blasting system consisted of a CO_2_ cylinder, a pressure reducing valve, a high-pressure solenoid valve, a pressure gauge, and an explosion-proof inflation device, this device was convenient for releasing a constant pressure to fracture the sample at the moment of blasting. Before conducting the experiment, check the air tightness of the CO_2_ blasting system, and then connect the CO_2_ cylinder to the gas blasting tube. Observe the pressure gauge, adjust the pressure reducing valve to 1.5MPa, and open the high-pressure solenoid valve to release CO_2_. We added the description in the **Experiment System** section.

Issue 8. The authors stated that ‘the grayscale is quantized from 256 levels into 64 levels’, however, the red and the blue color in original figures had been altered to all white color, see Fig. 5. How did the authors consider the errors this degradation could incur?

*Response*:

Due to the abundance of images in Fig. 5, we had to resize the images to allow for a side-by-side comparison and analysis. Here, we present the grayscale image and contour plot. In the contour plot, it is evident that not all red and blue color in original figures turn completely white; instead, color with similar grayscale values may create an illusion. Therefore, we adopted a comprehensive approach involving both grayscale and texture feature analysis.

Figure 3. Original Grayscale Image

Figure 4. Contour Plot

Thank you again for your selfless review of the manuscript and for providing references with high relevance and learning value. The review comments sincerely and carefully pointed out the main problems existing in the paper. By studying the references provided and carefully revising them, the overall level of the paper has been greatly improved. The continuous improvement of the paper makes our research more conducive to the reading of a wide range of readers and can inspire new ideas for relevant research.

**Reviewer #8**

This article uses gray and texture analysis methods, combined with CO_2_ blasting, and through theoretical analysis and laboratory experiments, it shows the process of CO_2_ blasting-induced coal rock fracture, and obtains the precursory characteristics of cracks from initiation to expansion. The scientific nature of the work is good. I think this research is meaningful, but this article needs to be minor revisions so that it can be published.

Thank you very much for reviewing the manuscript and giving us the opportunity to revise and improve its quality. We have revised the article in terms of technology and writing. Please review it.

Issue 1. Compared with the original manuscript, the grammatical problems of the revised manuscript have been greatly improved, but there are still some small problems that need to be modified.

*Response*:

Thank you for your review and valuable feedback. I'm glad to hear that the revised manuscript has shown significant improvement in terms of grammar. I will continue to work on addressing the remaining minor issues you have pointed out to ensure the paper's grammar is precise and fluent.

Issue 2. Previous studies on CO_2_ induced fracturing have done a lot of work. It is suggested that the author should evaluate objectively in the citation, and highlight the characteristics of the research and the difference from the existing research.

*Response*:

Thanks for your valuable comments, we have added relevant descriptions in the last paragraph of **Introduction** section. Please check it.

Issue 3. It is recommended to upload a clearer version of Figure 7 for publication.

*Response*:

Thanks for your valuable comments, we have re-uploaded the figure and modified the font size.

Issue 4. How many experiments were performed? Please add description in experiment section.

*Response*:

We conducted at least 5 experiments under each experimental condition. And typical results were selected for analysis. A description was added.

Figure 1. Some specimens

Issue 5. Were any saturation performed before any experiment?

*Response*:

No, they were not saturated. The reason was shown flowing:

As is well known, the presence of bubbles during the specimen-making process can reduce its strength. Some scholars have eliminated this effect by keeping specimens in water for 14 days, which is a very good method [1]. However, it is worth noting that the specimens we make are intended to approximate the properties of coal. Unlike rocks, coal has a moisture content that can seriously affect its strength and even combine with the organic components in coal [2]. Therefore, many scholars have conducted research on exhaust operations through vibration, static load application, and other methods during the production of specimens [3]. We not only used the above method in the process of making the specimens but also saved them through constant temperature and humidity experimental devices. In addition, this change in strength is reduced by applying a static load during the solidification process of the specimen.

Issue 6. Conclusion The description is too complicated, and it is suggested to reorganize the language.

*Response*:

Thanks for your valuable comments, we have modified the **Conclusion** section, and added a section for **Discussion**.

Issue 7. It is suggested that the last paragraph of the conclusion be re-summarized into a chapter and placed before the conclusion, which will be clearer. Just like this:

Discussion

“The main strain field grayscale and texture features parameters can quantitatively characterize the coal rock cracking and propagation during the CO_2_ blasting process and determine the times of coal rock cracking and propagation. The goal is to improve blasting permeability and gas extraction efficiency while avoiding environmental and safety issues caused by CO_2_ misuse. The study provides a theoretical basis for enhancing gas extraction from coal seams. However, due to the limitations of simulation experiment conditions, a limitation of this study is that it solely employed uniaxial loading. The findings can be extrapolated to scenarios involving the fracturing of shallowly buried coal and rock, as well as coal rock containing numerous vertical faults, residual coal pillars, inclined coal seam slopes, and analogous rock-like materials without confining pressure. Nonetheless, for a more extensive range of applications, it becomes imperative to account for confining pressure within a closed-box environment, alongside the ramifications of CO_2_ adsorption by coal. Additionally, the coal seam's adsorption characteristics, mechanical properties, and relevant conditions such as gas concentration should be considered to provide better theoretical guidance for CO_2_ blasting permeability enhancement technology.”

*Response*:

Thank you for your valuable comments. We have made modifications according to your suggestion. This change makes the article more organized.

Thank you again for your selfless review of the manuscript and for providing references with high relevance and learning value. The review comments sincerely and carefully pointed out the main problems existing in the paper. By studying the references provided and carefully revising them, the overall level of the paper has been greatly improved. The continuous improvement of the paper makes our research more conducive to the reading of a wide range of readers and can inspire new ideas for relevant research.

***References***

[1]	Tariq Z, Mahmoud M, Abdulraheem A, et al. An environment friendly approach to reduce the breakdown pressure of high strength unconventional rocks by cyclic hydraulic fracturing[J]. Journal of Energy Resources Technology, 2020, 142(4): 043002.

[2]	Yao Q, Chen T, Ju M, et al. Effects of water intrusion on mechanical properties of and crack propagation in coal[J]. Rock Mechanics and Rock Engineering, 2016, 49: 4699-4709.

[3]	Zhao B, Li S, Lin H, et al. Experimental Study of Influencing Factors on Mechanical Properties for New Coal-Like Materials[J]. Journal of Energy Engineering, 2023, 149(4): 04023022.

**Reviewer #9**

本研究重点研究了CO_2_注入过程中应变场云图像灰度和织构特征的演变。构建了CO_2_爆破引起的煤岩断裂压力模型和CO_2_爆破气体压力分布模型。定量研究了CO_2_爆破应变场的灰度特征，分析了织构特征参数随时间的变化规律。该研究旨在提高鼓风渗透性和气体提取效率，同时避免与二氧化碳滥用相关的环境和安全问题。虽然我发现了一些改善工作的问题，如下所示

Thank you very much for reviewing the manuscript and giving us the opportunity to revise and improve its quality. We have revised the article in terms of technology and writing. Please review it.

Issue 1. 手稿中使用的英语太差了，以至于无法理解一句话的含义。

*Response*:

Due to language constraints, there are many grammatical problems in our paper, which makes it difficult to understand. We apologize for this. We carefully revised the whole article after checking for grammar errors, incorrect words, and other problems. We have invited the AJE organization to proofread our paper; specifically, this organization ensures that native speakers of English linguistics have revised and proofread the whole article. Modifying the readability and logic of the paper can arouse the interest of a wide range of readers, make the article better show our phased achievements, and provide a new research method for relevant researchers, as shown in Figure 1.

Figure 1. Proof of proofreading

Issue 2. 手稿中提到的方程式都是已知的和预先存在的。在这方面也没有新的贡献。

*Response*:

Part of the theory and formulas in this study are based on the existing literature, and we have given the references.

Issue 3. 分析不足以验证结果。

*Response*:

Thank you for your valuable opinions. The conclusion of this paper is based on the experimental results. In previous studies, our team developed a small-size test platform, carried out some tests of parameters such as vibration, displacement, crack development width and length [1-5]. The fracture initiation pressure model and gas pressure distribution model proposed in this paper have been well verified in laboratory experiments. It may be that our problems in writing have caused you trouble. We have revised the **Results and discussion** section. Please check it.

Issue 4. CO_2_压裂与水力压裂在压裂压力和裂缝网络复杂性方面的比较如何？

*Response*:

Thanks for your valuable comments, we have added a comparison between CO_2_ fracturing and hydraulic fracturing in the initiation pressure and fracture network complexity in the **Introduction** section. Please check it.

Lu et al. [6] found that the fracture initiation pressure when fracturing shale with SC-CO_2_ was about 50.9% lower than that of hydraulic fracturing, and the fracture initiation pressure when fracturing sandstone was about 57.1% lower. CO_2_ blasting can create complex fractures network and avoid water blocking effects. The observed fracture aperture for CO_2_ blasting is much greater than that for hydraulic fracturing, the maximum fracture aperture for CO_2_ blasting is around 2-5 times greater than that for hydraulic fracturing under the same test conditions. The permeability test results show that the permeability of CO_2_ blasting is 190.43 times that of hydraulic fracturing [7].

Issue 5. 灰度和织构分析在确定煤岩开裂再展开前驱特征方面得出的结论是什么？

*Response*:

Thanks for your valuable comments, we have modified the **Conclusion** section, and added a section for **Discussion**.

We first constructed a gas pressure distribution model in the borehole and a CO_2_ blasting-induced coal rock fracture pressure model, and calculated the pressure distribution in the borehole under different initial pressure conditions. Then we carried out the CO_2_ blasting experiments with different initial pressures to obtain the main strain cloud maps of the sample surface. The GLCM was used to quantitatively study the evolution rules of grayscale and texture characteristics of the main strain field on the specimen surface during the experiment. Analysing the experimental data found that the texture feature parameters will mutate at 3ms and 9ms. Combined with the CO_2_ explosion gas pressure distribution model, when the initial pressure is 1.5MPa, the pressure in the borehole is not significantly increased at 3ms, the coal rock has a tendency to fracture. After the coal rock fractures, the gas escape and the change in the borehole volume made the model unsuitable for determining the crack propagation time, but the crack propagation time can be determined through the second mutation of the texture characteristic parameters. The model will continue to be improved in subsequent research.

Issue 6. 与CO_2_注入相关的研究的目的是什么？

*Response*:

Our main research is CO_2_ blasting. The introduction of research related to CO_2_ blasting is intended to inform the reader of this method of enhancing the permeability of coal seams. After that, we introduce a method to analyze and monitor the characteristics of coal rock cracking precursor by using gray and texture features.

Issue 7. 研究中如何获得应变场的灰度和织构参数？

*Response*:

To explain the technical process more clearly, **Image analysis methodology** has been added to the **Materials and methods** section.

Issue 8. 我没有看到任何新颖的东西可以提交给影响力较低的期刊考虑。

*Response*:

Thank you for taking the time out of your busy schedule to review our article. There are currently many studies on CO_2_ blasting, and we introduced many related studies in the introduction section. The current characterization of coal rock fracture states is mainly based on experimental characterization, such as CT scanning [8], nuclear magnetic resonance [9], 3D morphological scanning [10], and acoustic emission [11], with fewer quantitative analysis methods. In subsequent analysis, it is necessary to quantitatively study the evolution characteristics of strain field in coal rock fracture states.

Grayscale and texture parameters are vital to quantitatively describe image features and are widely applied in many fields, such as medicine, agriculture, and mining [12, 13]. The strain field can reflect the dynamic, unstable process of coal failure [14], and grayscale and texture features can accurately describe the fracturing stages of coal and rock, as previous studies have shown. Xu studied the crack initiation and propagation process in granite using the texture feature parameters of images and verified its feasibility in reflecting the rock failure mechanism [15]. Sun established a damage model based on texture features and studied the generation and propagation process of microcracks in coal under uniaxial compression [16]. By quantifying the strain field, Zhang studied the evolution of the grayscale and texture parameters in the strain field during the compressive deformation of fractured sandstone specimens [17].

Therefore, in view of the current insufficient quantitative research on the CO_2_ blasting-induced strain field, we propose a new method based on image feature analysis.

Thank you again for your selfless review of the manuscript and for providing references with high relevance and learning value. The review comments sincerely and carefully pointed out the main problems existing in the paper. By studying the references provided and carefully revising them, the overall level of the paper has been greatly improved. The continuous improvement of the paper makes our research more conducive to the reading of a wide range of readers and can inspire new ideas for relevant research.

***References***

[1]	Wang K, Pan H, Zhang T, et al. Experimental study on the radial vibration characteristics of a coal briquette in each stage of its life cycle under the action of CO_2_ gas explosion[J]. Fuel, 2022, 320: 123922.

[2]	Wang K, Pan H, Zhang T. Experimental study of prefabricated crack propagation in coal briquettes under the action of a CO_2_ gas explosion[J]. ACS omega, 2021, 6(38): 24462-24472.

[3]	Pan H, Li J, Zhang T, et al. Study on crack propagation of the CO_2_ presplitting blasting empty hole effect in coal seam[J]. Energy Science & Engineering, 2020, 8(11): 3898-3908.

[4]	Li J, Pan H, Gong W, et al. Study on rock breaking characteristics of stress wave and blast-induced gas during CO_2_ gas blasting[J]. Journal of Petroleum Science and Engineering, 2022, 219: 111142.

[5]	Pan H, Qin B, Zhang T, et al. Influence of CO_2_ gas explosion on moulded-coal crack propagation under static load[J]. Journal of Xi'an University of Science andTechnology, 2022, 42(2),251-259. DOI:10.13800/j.cnki.xakjdxxb.2022.0208

[6]	Lu YY, Liao Y, Tang JR, Zhang XW, Han SB, Ling YF. Experimental study on fracture initiation pressure and morphology in shale using supercritical CO_2_ fracturing. J. China Coal Soc. 2018; 43(1), 175-180.

[7]	Jia B, Tsau JS, Barati R. A review of the current progress of CO_2_ injection EOR and carbon storage in shale oil reservoirs. Fuel. 2019; 236, 404-427.

[8]	Yang B, Wang HZ, Wang B, Shen ZH, Zheng Y, Jia ZY, et al. Digital quantification of fracture in full-scale rock using micro-CT images: A fracturing experiment with N_2_ and CO_2_. J. Pet. Sci. Eng. 2021; 196, 107682.

[9]	Sun HQ, Wang HT, Lun ZM. Effect of permeability and fractures on oil mobilization of unconventional resources during CO_2_ EOR using nuclear magnetic resonance. Sci. Rep. 2021; 11, 1-6.

[10]	Yang JF, Lian HJ, Li L. Fracturing in coals with different fluids: an experimental comparison between water, liquid CO_2_, and supercritical CO_2_. Sci. Rep. 2020; 10, 1-15.

[11]	Li HL, Zhou L, Lu YY, Yan FZ, Zhou JK, Tang JR. Influence of supercritical CO_2_ saturation on the failure process of hot dry rock with acoustic emission monitoring. Powder Technol. 2020; 374, 241-249.

[12]	Orlhac F, Soussan M, Maisonobe J A, et al. Tumor texture analysis in 18F-FDG PET: relationships between texture parameters, histogram indices, standardized uptake values, metabolic volumes, and total lesion glycolysis. J. Nucl. Med. 2014; 55(3), 414-422.

[13]	Hodgdon T, McInnes M D F, Schieda N, et al. Can quantitative CT texture analysis be used to differentiate fat-poor renal angiomyolipoma from renal cell carcinoma on unenhanced CT images? Radiology. 2015; 276(3), 787-796.

[14]	Shaojie Chen, Faxin Li, Dawei Yin, et al. Experimental study on deformation failure characteristics of limestone−coal composite with different rock-coal height ratios. Journal of Central South University (Science and Technology). 2023; 54(06), 2459-2472.

[15]	Xu J, Liu F, Chen Z, et al. Digital features of main constituents in granite during crack initiation and propagation. Eng. Geol. 2017; 225, 96-102.

[16]	Sun C, Cao S, Li Y. Mesomechanics coal experiment and an elastic-brittle damage model based on texture features. Int. J. Min. Sci. Technol. 2018; 28(4), 639-647.

[17]	Zhang K, Zhang K. Gray and texture features of strain field for fractured sandstone during failure process[J]. J. China Coal Soc. 2021; 46(4), 1253-1262.

**Reviewer #10**

In this study, the model of coal rock fracture pressure caused by CO_2_ blasting and the model of gas pressure distribution caused by CO_2_ blasting are constructed. The gray-scale characteristics of CO_2_ blasting strain field are quantitatively studied, and the variation of texture parameters with time is analyzed. The aim is to improve blast permeability and gas extraction efficiency, while reducing environmental and safety concerns associated with CO_2_ abuse. Therefore, this study is not very innovative and I do not think it is suitable for publication in PLOS ONE journal. The following suggestions are given to the author for reference:

Thank you very much for reviewing the manuscript and giving us the opportunity to revise and improve its quality. We have revised the article in terms of technology and writing. Please review it.

Issue 1. The language in the manuscript is difficult to understand, and it is impossible to read the meaning expressed in the article, and it is recommended to improve the use of language.

*Response*:

Due to language constraints, there are many grammatical problems in our paper, which makes it difficult to understand. We apologize for this. We carefully revised the whole article after checking for grammar errors, incorrect words, and other problems. We have invited the AJE organization to proofread our paper; specifically, this organization ensures that native speakers of English linguistics have revised and proofread the whole article. Modifying the readability and logic of the paper can arouse the interest of a wide range of readers, make the article better show our phased achievements, and provide a new research method for relevant researchers, as shown in Figure 1.

Figure 1. Proof of proofreading

Issue 2. Most of the references in this paper are Chinese scholars, so we should make appropriate reference to foreign scholars to enrich the basic research of this article.

*Response*:

Thank you for your diligent review and valuable suggestions. We have replaced some references.

Indeed, a substantial portion of the references cited in my paper are derived from Chinese literature or authored by researchers based in China.

China stands out globally as one of the countries most severely affected by coal and gas outburst disasters. The gas geological reserves within 2000 m of burial depth are estimated to be approximately 36.81 trillion cubic meters, comparable to the onshore conventional natural gas reserves of 38 trillion cubic meters. However, due to the low permeability of coal seams (2-3 orders of magnitude lower than in the United States), extracting gas from these coal seams presents significant challenges, posing a serious threat to mine safety and production.

The focus of my paper is on investigating a method to enhance coal seam permeability, particularly applicable to regions with low coal seam permeability, such as many coalfields in China. So most of the references are from Chinese authors. These references from Chinese authors are from highly influential journals.

Issue 3. The model constructed and the formula used in this article are already ubiquitous and not innovative.

*Response*:

Part of the theory and formulas in this study are based on the existing literature, and we have given the references.

Issue 4. The content of the article is single, there are few diagrams, and it is not convincing.

*Response*:

Thank you for taking the time out of your busy schedule to review our article. We have revised the content and format of the article.

Issue 5. The analysis of the conclusions obtained in the article is too simple to prove the topic of the article, and it is recommended to further condense the conclusions.

*Response*:

Thanks for your valuable comments, we have modified the **Conclusion** section, and added a section for **Discussion**.

Issue 6. How to obtain the grayscale and texture parameters of the strain field in the study are not reflected in the paper, and it is recommended that the authors add further.

*Response*:

To explain the technical process more clearly, **Image analysis methodology** has been added to the **Materials and methods** section.

Issue 7. The results are not of publication value, as conclusions are usually not general and some conclusions are debatable or even wrong.

*Response*:

Thank you for your valuable opinions. The conclusion of this paper is based on the experimental results. In previous studies, our team developed a small-size test platform, carried out some tests of parameters such as vibration, displacement, crack development width and length [1-5]. The fracture initiation pressure model and gas pressure distribution model proposed in this paper have been well verified in laboratory experiments. It may be that our problems in writing have caused you trouble. We have revised the **Conclusion** section. Please check it.

Issue 8. The overall content of the article is not innovative and of poor quality, and it is recommended to turn to journals with low impact.

Thank you for taking the time out of your busy schedule to review our article. There are currently many studies on CO_2_ blasting, and we introduced many related studies in the introduction section. The current characterization of coal rock fracture states is mainly based on experimental characterization, such as CT scanning [6], nuclear magnetic resonance [7], 3D morphological scanning [8], and acoustic emission [9], with fewer quantitative analysis methods. In subsequent analysis, it is necessary to quantitatively study the evolution characteristics of strain field in coal rock fracture states.

Grayscale and texture parameters are vital to quantitatively describe image features and are widely applied in many fields, such as medicine, agriculture, and mining [10, 11]. The strain field can reflect the dynamic, unstable process of coal failure [12], and grayscale and texture features can accurately describe the fracturing stages of coal and rock, as previous studies have shown. Xu studied the crack initiation and propagation process in granite using the texture feature parameters of images and verified its feasibility in reflecting the rock failure mechanism [13]. Sun established a damage model based on texture features and studied the generation and propagation process of microcracks in coal under uniaxial compression [14]. By quantifying the strain field, Zhang studied the evolution of the grayscale and texture parameters in the strain field during the compressive deformation of fractured sandstone specimens [15].

Therefore, in view of the current insufficient quantitative research on the CO_2_ blasting-induced strain field, we propose a new method based on image feature analysis.

Thank you again for your selfless review of the manuscript and for providing references with high relevance and learning value. The review comments sincerely and carefully pointed out the main problems existing in the paper. By studying the references provided and carefully revising them, the overall level of the paper has been greatly improved. The continuous improvement of the paper makes our research more conducive to the reading of a wide range of readers and can inspire new ideas for relevant research.

***References***

[1]	Wang K, Pan H, Zhang T, et al. Experimental study on the radial vibration characteristics of a coal briquette in each stage of its life cycle under the action of CO_2_ gas explosion[J]. Fuel, 2022, 320: 123922.

[2]	Wang K, Pan H, Zhang T. Experimental study of prefabricated crack propagation in coal briquettes under the action of a CO_2_ gas explosion[J]. ACS omega, 2021, 6(38): 24462-24472.

[3]	Pan H, Li J, Zhang T, et al. Study on crack propagation of the CO_2_ presplitting blasting empty hole effect in coal seam[J]. Energy Science & Engineering, 2020, 8(11): 3898-3908.

[4]	Li J, Pan H, Gong W, et al. Study on rock breaking characteristics of stress wave and blast-induced gas during CO_2_ gas blasting[J]. Journal of Petroleum Science and Engineering, 2022, 219: 111142.

[5]	Pan H, Qin B, Zhang T, et al. Influence of CO_2_ gas explosion on moulded-coal crack propagation under static load[J]. Journal of Xi'an University of Science andTechnology, 2022, 42(2),251-259. DOI:10.13800/j.cnki.xakjdxxb.2022.0208

[6]	Yang B, Wang HZ, Wang B, Shen ZH, Zheng Y, Jia ZY, et al. Digital quantification of fracture in full-scale rock using micro-CT images: A fracturing experiment with N_2_ and CO_2_. J. Pet. Sci. Eng. 2021; 196, 107682.

[7]	Sun HQ, Wang HT, Lun ZM. Effect of permeability and fractures on oil mobilization of unconventional resources during CO_2_ EOR using nuclear magnetic resonance. Sci. Rep. 2021; 11, 1-6.

[8]	Yang JF, Lian HJ, Li L. Fracturing in coals with different fluids: an experimental comparison between water, liquid CO_2_, and supercritical CO_2_. Sci. Rep. 2020; 10, 1-15.

[9]	Li HL, Zhou L, Lu YY, Yan FZ, Zhou JK, Tang JR. Influence of supercritical CO_2_ saturation on the failure process of hot dry rock with acoustic emission monitoring. Powder Technol. 2020; 374, 241-249.

[10]	Orlhac F, Soussan M, Maisonobe J A, et al. Tumor texture analysis in 18F-FDG PET: relationships between texture parameters, histogram indices, standardized uptake values, metabolic volumes, and total lesion glycolysis. J. Nucl. Med. 2014; 55(3), 414-422.

[11]	Hodgdon T, McInnes M D F, Schieda N, et al. Can quantitative CT texture analysis be used to differentiate fat-poor renal angiomyolipoma from renal cell carcinoma on unenhanced CT images? Radiology. 2015; 276(3), 787-796.

[12]	Shaojie Chen, Faxin Li, Dawei Yin, et al. Experimental study on deformation failure characteristics of limestone−coal composite with different rock-coal height ratios. Journal of Central South University (Science and Technology). 2023; 54(06), 2459-2472.

[13]	Xu J, Liu F, Chen Z, et al. Digital features of main constituents in granite during crack initiation and propagation. Eng. Geol. 2017; 225, 96-102.

[14]	Sun C, Cao S, Li Y. Mesomechanics coal experiment and an elastic-brittle damage model based on texture features. Int. J. Min. Sci. Technol. 2018; 28(4), 639-647.

[15]	Zhang K, Zhang K. Gray and texture features of strain field for fractured sandstone during failure process[J]. J. China Coal Soc. 2021; 46(4), 1253-1262.

**Reviewer #11**

The content of the article is complete and the structure is clear, and it has been greatly improved after revision. I would like to ask the author to revise some details, and suggest that it be published after minor revision.

Thank you very much for reviewing the manuscript and giving us the opportunity to revise and improve its quality. We have revised the article in terms of technology and writing. Please review it.

Issue 1. Line 255, the abbreviation ASM, it is recommended that the full name be Angular Second Moment instead of energy, which can be confusing for readers outside the field.

*Response*:

We apologize for the incorrect expression. We have revised it in the manuscript. Please check it.

Issue 2. Line 256, Correlation is abbreviated as CON, while CORRLN is written in Table 2, which needs to be unified.

*Response*:

We apologize for the error. We have unified the abbreviations in Table 2 with those in the article.

Issue 3. In the manuscript, we can see that the author sets three groups of samples, and only analyzes the case of 1.5MPa in the results and discussion. Why choose the case of 1.5MPa for analysis? Why not choose 0.5Mpa or 1.0MPa? I think the other two groups should be properly analyzed even as control groups. Please reply.

*Response*:

Our initial pressure was selected based on the similarity ratio ladder according to the actual situation of the field engineering test. There are some explanations as follows:

To explore the characteristics of fracturing pressure, initial pressures of 0.5 MPa, 1.0 MPa, and 1.5 MPa were selected to carry out the tests. Other scholars have also used these three typical pressures [1, 2]. Scholars have carried out experiments of CO_2_ blasting in the range of 1.2 MPa - 3.0 MPa. When the pressure is 1.2 MPa - 1.8 MPa, it is the crack initiation stage of the borehole fracture zone [3]. Therefore, we focused on the analysis of experimental data under 1.5 MPa conditions.

Due to the limitation of test conditions, the test effect is best when the initial air pressure is 1.5MPa, so we choose to focus on analyzing the data under this pressure. Figure 1 is strain field of the other two groups.

(a) P=0.5 MPa

(b) P=1.0 MPa

Figure 1. The maximum principal strain distribution.

We have added explanations in **Discussion** section.

The limitations of this study are as follows: (1) This study is a 2-D model to explore the characteristics of the crushing/fractured/elastic zone area. However, previous studies have shown that the formation of fractures by CO_2_ fracturing may be a 3-D fractured network with angles and has a certain degree of complexity. (2) The effectiveness of gas extraction in coal seams is an important indicator for evaluating fracturing. In this study, the development of fractures, changes in mechanical parameters, and the morphology of damaged areas were mainly studied. The effectiveness of gas extraction should also be considered in the future. (3) Coal is a heterogeneous material, and the joint surface and initial pore structure may affect the fracture failure area. There is insufficient research on heterogeneous materials fracture.

Given the limitations of the current research, we are currently improving the experimental equipment and carrying out some related work:

(1) On the basis of the existing 2-D model, the thickness of the samples has been increased, and more fracturing parameters in the space have been obtained by adding more sensors in the 3-D space ahead of the fracturing.

(2) By applying CH_4_ to the system before conducting the experiment to saturate the sample. CO_2_ and CH_4_ concentrations are measured before and after fracturing to evaluate the effectiveness.

(3) Samples using different materials are being made, and the development characteristics of cracks on heterogeneous interfaces are being studied by designing and adding gangue layers, faults, etc.

(4) On the basis of the application of temperature control systems, more temperature sensors and stress sensors are added inside the sample to study the impact of thermal stress generated by CO_2_ fracturing process heat absorption on coal rock failure. It is expected to obtain the temperature field changes during the whole fracturing process.

(5) By adding more non-destructive detection equipment, including but not limited to electronic probes, borehole peepers, non-destructive scanning equipment, etc. The development morphology of fractures can be obtained in real-time, and a 3-D simulated fracture network model can be established.

Thank you again for your selfless review of the manuscript and for providing references with high relevance and learning value. The review comments sincerely and carefully pointed out the main problems existing in the paper. By studying the references provided and carefully revising them, the overall level of the paper has been greatly improved. The continuous improvement of the paper makes our research more conducive to the reading of a wide range of readers and can inspire new ideas for relevant research.

***References***

[1]	Pan H, Li J, Zhang T, et al. Study on crack propagation of the CO_2_ presplitting blasting empty hole effect in coal seam[J]. Energy Science & Engineering, 2020, 8(11): 3898-3908.

[2]	Wang K, Pan H, Zhang T. Experimental study of prefabricated crack propagation in coal briquettes under the action of a CO_2_ gas explosion[J]. ACS omega, 2021, 6(38): 24462-24472.

[3]	Wen H, Li Z B, Wang Z, et al. Experiment on the liquid CO_2_ fracturing process for increasing permeability and the characteristics of crack propagation in coal seam [J]. Journal of China Coal Society, 2016, 41(11):2793-2799. DOI: 10.13225/j.cnki.jccs.2016.0124.

**Reviewer #12**

In this study, a series of CO_2_ blasting tests were conducted on coal-like material to investigate the precursory features of coal rock cracking and crack propagation. The tests were carried out under different initial gas pressure conditions (0.5 MPa, 1.0 MPa, and 1.5 MPa). The non-contact full-strain field measurement analysis system was used to analyze the evolution law of strain field and texture features. The manuscript can be accepted if the authors consider the following suggestions:

Thank you very much for reviewing the manuscript and giving us the opportunity to revise and improve its quality. We have revised the article in terms of technology and writing. Please review it.

Issue 1. The authors have provided a comprehensive review of previous research on CO_2_ blasting in the second paragraph of the Introduction (Line 52-76). However, the literature review is too lengthy and should be condensed to highlight the similarities and differences.

*Response*:

Thanks for your valuable comments, we have streamlined the literature review section and deleted or replaced lengthy sentences.

Issue 2. If the theory and calculation formulas in this study are based on existing literature, it is recommended to include references.

*Response*:

Part of the theory and formulas in this study are based on the existing literature, and we have given the references.

Issue 3. It is recommended to include a sample diagram and specify the size of the gas blast tube or the sample notch.

*Response*:

Thank you for your valuable comments. In Figure 4, we added two photos taken in the experiment, marking the analysis area and notch size respectively.

Issue 4. What is the rationale for establishing different initial gas pressure conditions? Does it hold specific practical engineering significance?

*Response*:

Our initial pressure was selected based on the similarity ratio ladder according to the actual situation of the field engineering test. There are some explanations as follows:

To explore the characteristics of fracturing pressure, initial pressures of 0.5 MPa, 1.0 MPa, and 1.5 MPa were selected to carry out the tests. Other scholars have also used these three typical pressures [1, 2]. Scholars have carried out experiments of CO_2_ blasting in the range of 1.2 MPa - 3.0 MPa. When the pressure is 1.2 MPa - 1.8 MPa, it is the crack initiation stage of the borehole fracture zone [3]. Therefore, we focused on the analysis of experimental data under 1.5 MPa conditions.

Issue 5. In Figure 5, the principal strain field, grayscale image, and gray histogram exhibit a clear trend. How do the results of the other two groups (when the initial gas pressure is 0.5 MPa and 1.0 MPa) compare?

*Response*:

Due to the limitation of test conditions, the test effect is best when the initial air pressure is 1.5MPa, so we choose to focus on analyzing the data under this pressure. Figure 1 is strain field of the other two groups.

(a) P=0.5 MPa

(b) P=1.0 MPa

Figure 1. The maximum principal strain distribution.

We have added explanations in **Discussion** section.

The limitations of this study are as follows: (1) This study is a 2-D model to explore the characteristics of the crushing/fractured/elastic zone area. However, previous studies have shown that the formation of fractures by CO_2_ fracturing may be a 3-D fractured network with angles and has a certain degree of complexity. (2) The effectiveness of gas extraction in coal seams is an important indicator for evaluating fracturing. In this study, the development of fractures, changes in mechanical parameters, and the morphology of damaged areas were mainly studied. The effectiveness of gas extraction should also be considered in the future. (3) Coal is a heterogeneous material, and the joint surface and initial pore structure may affect the fracture failure area. There is insufficient research on heterogeneous materials fracture.

Given the limitations of the current research, we are currently improving the experimental equipment and carrying out some related work:

(1) On the basis of the existing 2-D model, the thickness of the samples has been increased, and more fracturing parameters in the space have been obtained by adding more sensors in the 3-D space ahead of the fracturing.

(2) By applying CH_4_ to the system before conducting the experiment to saturate the sample. CO_2_ and CH_4_ concentrations are measured before and after fracturing to evaluate the effectiveness.

(3) Samples using different materials are being made, and the development characteristics of cracks on heterogeneous interfaces are being studied by designing and adding gangue layers, faults, etc.

(4) On the basis of the application of temperature control systems, more temperature sensors and stress sensors are added inside the sample to study the impact of thermal stress generated by CO_2_ fracturing process heat absorption on coal rock failure. It is expected to obtain the temperature field changes during the whole fracturing process.

(5) By adding more non-destructive detection equipment, including but not limited to electronic probes, borehole peepers, non-destructive scanning equipment, etc. The development morphology of fractures can be obtained in real-time, and a 3-D simulated fracture network model can be established.

Thank you again for your selfless review of the manuscript and for providing references with high relevance and learning value. The review comments sincerely and carefully pointed out the main problems existing in the paper. By studying the references provided and carefully revising them, the overall level of the paper has been greatly improved. The continuous improvement of the paper makes our research more conducive to the reading of a wide range of readers and can inspire new ideas for relevant research.

***References***

[1]	Pan H, Li J, Zhang T, et al. Study on crack propagation of the CO_2_ presplitting blasting empty hole effect in coal seam[J]. Energy Science & Engineering, 2020, 8(11): 3898-3908.

[2]	Wang K, Pan H, Zhang T. Experimental study of prefabricated crack propagation in coal briquettes under the action of a CO_2_ gas explosion[J]. ACS omega, 2021, 6(38): 24462-24472.

[3]	Wen H, Li Z B, Wang Z, et al. Experiment on the liquid CO_2_ fracturing process for increasing permeability and the characteristics of crack propagation in coal seam [J]. Journal of China Coal Society, 2016, 41(11):2793-2799. DOI: 10.13225/j.cnki.jccs.2016.0124

**Reviewer #13**

I can’t see any novelties and it can be submitted to lower impact journals for consideration. The manuscript is not suitable for consideration in its present format. In this paper, I have found some issues towards the betterment of the work which are listed below:

Issue 1. English used in the manuscript is very poor to understand the meaning of one sentence.

*Response*:

Due to language constraints, there are many grammatical problems in our paper, which makes it difficult to understand. We apologize for this. We carefully revised the whole article after checking for grammar errors, incorrect words, and other problems. We have invited the AJE organization to proofread our paper; specifically, this organization ensures that native speakers of English linguistics have revised and proofread the whole article. Modifying the readability and logic of the paper can arouse the interest of a wide range of readers, make the article better show our phased achievements, and provide a new research method for relevant researchers, as shown in Figure 1.

Figure 1. Proof of proofreading

Issue 2. The equations mentioned in the manuscript are all known and pre-existing. Novel contribution in respect to that is also missing.

*Response*:

Part of the theory and formulas in this study are based on the existing literature, and we have given the references.

Issue 3. Analysis is not sufficient to validate the results.

*Response*:

Thanks for your valuable comments, we have modified the **Results and discussion** section.

In previous studies, our team developed a small-size test platform, carried out some tests of parameters such as vibration, displacement, crack development width and length [1-5]. The fracture initiation pressure model and gas pressure distribution model proposed in this paper have been well verified in laboratory experiments.

We first constructed a gas pressure distribution model in the borehole and a CO_2_ blasting-induced coal rock fracture pressure model, and calculated the pressure distribution in the borehole under different initial pressure conditions. Then we carried out the CO_2_ blasting experiments with different initial pressures to obtain the main strain cloud maps of the sample surface. The GLCM was used to quantitatively study the evolution rules of grayscale and texture characteristics of the main strain field on the specimen surface during the experiment. Analysing the experimental data found that the texture feature parameters will mutate at 3ms and 9ms. Combined with the CO_2_ explosion gas pressure distribution model, when the initial pressure is 1.5MPa, the pressure in the borehole is not significantly increased at 3ms, the rock has a tendency to fracture. After the rock fractures, the gas escape and the change in the borehole volume made the model unsuitable for determining the crack propagation time, but the crack propagation time can be determined through the second mutation of the texture characteristic parameters. The model will continue to be improved in subsequent research.

Issue 4. A comprehensive comparative analysis is missing to proof the outperformance of the proposed method.

*Response*:

To explain the technical process more clearly, **Image analysis methodology** has been added to the **Materials and methods** section.

The current characterization of coal rock fracture states is mainly based on experimental characterization, such as CT scanning [6], nuclear magnetic resonance [7], 3D morphological scanning [8], and acoustic emission [9], with fewer quantitative analysis methods. Therefore, we propose a method to analyze the change of strain field in coal rock during CO_2_ blasting. We have added a description in the **Introduction** section.

Issue 5. Benchmark testing as well as statistical analysis of the results is also missing in the work.

*Response*:

We conducted at least 5 experiments under each experimental condition. And typical results were selected for analysis. A description was added.

Figure 2. Some specimens.

Issue 6. Author has to perform more detail comparative analysis with the recent state-of-the-art method to validate the results.

*Response*:

Thank you for your valuable opinions. The questions you raised are exactly what we want to study in depth in the future. In previous studies, our team developed a small-size test platform, carried out some tests of parameters such as vibration, displacement, crack development width and length [10-15]. The fracture initiation pressure model and gas pressure distribution model proposed in this paper have been well verified in laboratory experiments. It may be that our problems in writing have caused you trouble. We have revised the **Results and discussion** section. Please check it.

We found some similar CO_2_ fracturing test equipment in the laboratory.

Table 1 Differences and verification with other experimental systems

| Authors | Specimen/material | Size/cm | System | The similarity in the results |
| --- | --- | --- | --- | --- |
| Ishida T, et al. [10] | granite | 17*17*17 |  | Small-size samples were easy to form through cracks, and the cracks were complex. |
| Ranjith P G, et al. [11] | coal-like materials | d=3.8  h=7.6 |  | With the CO_2_ pressure, the radial strain of coal increased, and the direction of the strain field inclined. |
| Kizaki A, et al. [12] | granite, tuff | 15*15*15 |  | CO_2_ fracturing was more likely to form fracture networks than hydraulic fracturing. |
| Zhou J, et al. [13] | shale | d=10  h=20 |  | CO_2_ fracturing was more effective, and more complex cracks were formed. |
| Zhang X, et al. [14] | shale | 20*20*20 |  | Increased injection pressure made material failure more obvious. The crack occurs at the weak position near the drill hole. |
| Wang L, el al. [15] | concrete | 20*20*20 |  | The fracture plane was rough, and the crack development was random, under low confining pressure and small stress difference. |

Thank you again for your selfless review of the manuscript and for providing references with high relevance and learning value. The review comments sincerely and carefully pointed out the main problems existing in the paper. By studying the references provided and carefully revising them, the overall level of the paper has been greatly improved. The continuous improvement of the paper makes our research more conducive to the reading of a wide range of readers and can inspire new ideas for relevant research.

***References***

[1]	Wang K, Pan H, Zhang T, et al. Experimental study on the radial vibration characteristics of a coal briquette in each stage of its life cycle under the action of CO_2_ gas explosion[J]. Fuel, 2022, 320: 123922.

[2]	Wang K, Pan H, Zhang T. Experimental study of prefabricated crack propagation in coal briquettes under the action of a CO_2_ gas explosion[J]. ACS omega, 2021, 6(38): 24462-24472.

[3]	Pan H, Li J, Zhang T, et al. Study on crack propagation of the CO2 presplitting blasting empty hole effect in coal seam[J]. Energy Science & Engineering, 2020, 8(11): 3898-3908.

[4]	Li J, Pan H, Gong W, et al. Study on rock breaking characteristics of stress wave and blast-induced gas during CO_2_ gas blasting[J]. Journal of Petroleum Science and Engineering, 2022, 219: 111142.

[5]	Pan H, Qin B, Zhang T, et al. Influence of CO_2_ gas explosion on moulded-coal crack propagation under static load[J]. Journal of Xi'an University of Science andTechnology, 2022, 42(2),251-259. DOI:10.13800/j.cnki.xakjdxxb.2022.0208

[6]	Sampath K, Perera M, Ranjith PG, Matthai SK, Yin LD. Qualitative and quantitative evaluation of the alteration of micro-fracture characteristics of supercritical CO_2_-interacted coal. J. Supercrit. Fluids. 2019; 147, 90-101.

[7]	Sun HQ, Wang HT, Lun ZM. Effect of permeability and fractures on oil mobilization of unconventional resources during CO_2_ EOR using nuclear magnetic resonance. Sci. Rep. 2021; 11, 1-6.

[8]	Yang JF, Lian HJ, Li L. Fracturing in coals with different fluids: an experimental comparison between water, liquid CO_2_, and supercritical CO_2_. Sci. Rep. 2020; 10, 1-15.

[9]	Li HL, Zhou L, Lu YY, Yan FZ, Zhou JK, Tang JR. Influence of supercritical CO_2_ saturation on the failure process of hot dry rock with acoustic emission monitoring. Powder Technol. 2020; 374, 241-249.

[10]	Ishida T, Aoyagi K, Niwa T, et al. Acoustic emission monitoring of hydraulic fracturing laboratory experiment with supercritical and liquid CO_2_[J]. Geophysical Research Letters, 2012, 39(16).

[11]	Ranjith P G, Perera M S A. A new triaxial apparatus to study the mechanical and fluid flow aspects of carbon dioxide sequestration in geological formations[J]. Fuel, 2011, 90(8): 2751-2759.

[12]	Kizaki A, Tanaka H, Ohashi K, et al. Hydraulic fracturing in Inada granite and Ogino tuff with super critical carbon dioxide[C]//ISRM International Symposium-Asian Rock Mechanics Symposium. ISRM, 2012: ISRM-ARMS7-2012-109.

[13]	Zhou J, Liu G, Jiang Y, et al. Supercritical carbon dioxide fracturing in shale and the coupled effects on the permeability of fractured shale: An experimental study[J]. Journal of Natural Gas Science and Engineering, 2016, 36: 369-377.

[14]	Zhang X, Lu Y, Tang J, et al. Experimental study on fracture initiation and propagation in shale using supercritical carbon dioxide fracturing[J]. Fuel, 2017, 190: 370-378.

[15]	Wang L, Yao B, Xie H, et al. Experimental investigation of injection-induced fracturing during supercritical CO_2_ sequestration[J]. International Journal of Greenhouse Gas Control, 2017, 63: 107-117.

**Reviewer #14**

The idea is not original enough and the amount of experimentation reflects too little. In adequate data analysis and presentation, also the structure of the article still needs to be adjusted. After our careful consideration, it does not fit PLOS ONE's position.

*Response*:

Thank you for taking the time out of your busy schedule to review our article. There are currently many studies on CO_2_ blasting, and we introduced many related studies in the introduction section. The current characterization of coal rock fracture states is mainly based on experimental characterization, such as CT scanning [1], nuclear magnetic resonance [2], 3D morphological scanning [3], and acoustic emission [4], with fewer quantitative analysis methods. In subsequent analysis, it is necessary to quantitatively study the evolution characteristics of strain field in coal rock fracture states.

Grayscale and texture parameters are vital to quantitatively describe image features and are widely applied in many fields, such as medicine, agriculture, and mining [5, 6]. The strain field can reflect the dynamic, unstable process of coal failure [7], and grayscale and texture features can accurately describe the fracturing stages of coal and rock, as previous studies have shown. Xu studied the crack initiation and propagation process in granite using the texture feature parameters of images and verified its feasibility in reflecting the rock failure mechanism [8]. Sun established a damage model based on texture features and studied the generation and propagation process of microcracks in coal under uniaxial compression [9]. By quantifying the strain field, Zhang studied the evolution of the grayscale and texture parameters in the strain field during the compressive deformation of fractured sandstone specimens [10].

Therefore, in view of the current insufficient quantitative research on the CO_2_ blasting-induced strain field, we propose a new method based on image feature analysis.

The conduction of such a study has not been undertaken by any team yet. In previous studies, our team developed a small-size test platform, carried out some tests of parameters such as vibration, displacement, crack development width and length [11-15]. The fracture initiation pressure model and gas pressure distribution model proposed in this paper have been well verified in laboratory experiments.

Thank you again for your selfless review of the manuscript and for providing references with high relevance and learning value. The review comments sincerely and carefully pointed out the main problems existing in the paper. By studying the references provided and carefully revising them, the overall level of the paper has been greatly improved. The continuous improvement of the paper makes our research more conducive to the reading of a wide range of readers and can inspire new ideas for relevant research.

***References***

[1]	Yang B, Wang HZ, Wang B, Shen ZH, Zheng Y, Jia ZY, et al. Digital quantification of fracture in full-scale rock using micro-CT images: A fracturing experiment with N_2_ and CO_2_. J. Pet. Sci. Eng. 2021; 196, 107682.

[2]	Sun HQ, Wang HT, Lun ZM. Effect of permeability and fractures on oil mobilization of unconventional resources during CO_2_ EOR using nuclear magnetic resonance. Sci. Rep. 2021; 11, 1-6.

[3]	Yang JF, Lian HJ, Li L. Fracturing in coals with different fluids: an experimental comparison between water, liquid CO_2_, and supercritical CO_2_. Sci. Rep. 2020; 10, 1-15.

[4]	Li HL, Zhou L, Lu YY, Yan FZ, Zhou JK, Tang JR. Influence of supercritical CO_2_ saturation on the failure process of hot dry rock with acoustic emission monitoring. Powder Technol. 2020; 374, 241-249.

[5]	Orlhac F, Soussan M, Maisonobe J A, et al. Tumor texture analysis in 18F-FDG PET: relationships between texture parameters, histogram indices, standardized uptake values, metabolic volumes, and total lesion glycolysis. J. Nucl. Med. 2014; 55(3), 414-422.

[6]	Hodgdon T, McInnes M D F, Schieda N, et al. Can quantitative CT texture analysis be used to differentiate fat-poor renal angiomyolipoma from renal cell carcinoma on unenhanced CT images? Radiology. 2015; 276(3), 787-796.

[7]	Shaojie Chen, Faxin Li, Dawei Yin, et al. Experimental study on deformation failure characteristics of limestone−coal composite with different rock-coal height ratios. Journal of Central South University (Science and Technology). 2023; 54(06), 2459-2472.

[8]	Xu J, Liu F, Chen Z, et al. Digital features of main constituents in granite during crack initiation and propagation. Eng. Geol. 2017; 225, 96-102.

[9]	Sun C, Cao S, Li Y. Mesomechanics coal experiment and an elastic-brittle damage model based on texture features. Int. J. Min. Sci. Technol. 2018; 28(4), 639-647.

[10]	Zhang K, Zhang K. Gray and texture features of strain field for fractured sandstone during failure process[J]. J. China Coal Soc. 2021; 46(4), 1253-1262.

[11]	Wang K, Pan H, Zhang T, et al. Experimental study on the radial vibration characteristics of a coal briquette in each stage of its life cycle under the action of CO_2_ gas explosion[J]. Fuel, 2022, 320: 123922.

[12]	Wang K, Pan H, Zhang T. Experimental study of prefabricated crack propagation in coal briquettes under the action of a CO_2_ gas explosion[J]. ACS omega, 2021, 6(38): 24462-24472.

[13]	Pan H, Li J, Zhang T, et al. Study on crack propagation of the CO_2_ presplitting blasting empty hole effect in coal seam[J]. Energy Science & Engineering, 2020, 8(11): 3898-3908.

[14]	Li J, Pan H, Gong W, et al. Study on rock breaking characteristics of stress wave and blast-induced gas during CO_2_ gas blasting[J]. Journal of Petroleum Science and Engineering, 2022, 219: 111142.

[15]	Pan H, Qin B, Zhang T, et al. Influence of CO_2_ gas explosion on moulded-coal crack propagation under static load[J]. Journal of Xi'an University of Science andTechnology, 2022, 42(2),251-259. DOI:10.13800/j.cnki.xakjdxxb.2022.0208

**Reviewer #15**

The paper entitled “Experimental study of precursory features of CO_2_ blasting-induced coal rock fracture based on grayscale and texture analysis” uses GLCM to extract the grayscale and texture features of the evolution of grayscale and textural features, quantitatively analyze the grayscale and texture features evolution of the main strain field on the sample surface during the CO_2_ blasting process, and identify the precursor characteristics of coal rock cracking and crack propagation. This research is interesting, and the developed test platform has specific application significance, which can provide a reference for the CO_2_ blasting mechanism. I think this manuscript can be accepted after the following revision.

Thank you very much for reviewing the manuscript and giving us the opportunity to revise and improve its quality. We have revised the article in terms of technology and writing. Please review it.

Issue 1. The axes and labels of some pictures are not clear.

*Response*:

Thank you for your suggestion. We have changed annotations and font sizes of all figures. Please check them.

Issue 2. Conclusion, nouns that appear previously should be consistent. For example, “Matlab” should be capitalized, or the previous text should be changed to lowercase.

*Response*:

We apologize for the error. We have modified it now.

Issue 3. Line 135-138, parameters should be italicized to be consistent with the formula.

*Response*:

Thanks for your careful review, we have changed the parameters to italics.

Issue 4. Line 149, the word “borepore” should be spelled incorrectly, “borehole” is used in the article, please check carefully. Line 227, Figure 5b should be revised to Fig 5b. Please check other similar errors.

*Response*:

Thank you for your diligent review and valuable suggestions, We have revised the question you raised.

Issue 5. When several quantities with units appear together, add the unit only after the last one. For example, “0.5 MPa, 1.0 MPa, and 1.5 MPa”, please check the full text and modify it.

*Response*:

Thanks for your careful review, We have changed “0.5 MPa, 1.0 MPa, and 1.5 MPa” to “0.5, 1.0, and 1.5 MPa”.

Thank you again for your selfless review of the manuscript and for providing references with high relevance and learning value. The review comments sincerely and carefully pointed out the main problems existing in the paper. By studying the references provided and carefully revising them, the overall level of the paper has been greatly improved. The continuous improvement of the paper makes our research more conducive to the reading of a wide range of readers and can inspire new ideas for relevant research.

**Reviewer #16**

I have reviewed an article “Experimental study of precursory features of CO_2_ blasting-induced coal rock fracture based on grayscale and texture analysis”. The manuscript falls within the scope of the journal. This manuscript can be considered for publication after the revision suggested below:

Thank you very much for reviewing the manuscript and giving us the opportunity to revise and improve its quality. We have revised the article in terms of technology and writing. Please review it.

Issue 1. Line 212: “Grayscale is an integer … brightness levels”. It does not reflect the advantages of grayscale analysis and it is recommended to modify it.

*Response*:

We have modified the description of grayscale in Line 220, please check it.

In digital image processing, grayscale is a commonly used image feature, which can be used to describe the brightness, contrast and texture of the image.

Issue 2. The font size in the image needs to be adjusted.

*Response*:

The font size, annotations, etc., of the images have been adjusted. Please check them.

Issue 3. Please check whether the parameters in the formula part are all annotated and correspond one to one.

*Response*:

Thank you for your valuable comments. We have checked all the formulas in the article.

Issue 4. Line 273: “As shown in Fig 7(b), from 0 ms to 3 ms …”. When describing this time period, only the last unit needs to be retained. The same error appears in this section, please check and correct it.

*Response*:

Thank you for your careful review. We have made a modification, please check it.

Issue 5. Conclusions needs to be streamlined.

*Response*:

Thanks for your valuable comments, we have modified the **Conclusion** section, and added a section for **Discussion**.

Thank you again for your selfless review of the manuscript and for providing references with high relevance and learning value. The review comments sincerely and carefully pointed out the main problems existing in the paper. By studying the references provided and carefully revising them, the overall level of the paper has been greatly improved. The continuous improvement of the paper makes our research more conducive to the reading of a wide range of readers and can inspire new ideas for relevant research.

**Reviewer #17**

Dear Editors and Authors:

I am honored to be able to review this paper. This paper studies the precursor information of precursory features of CO_2_ blasting-induced coal rock fracture based on grayscale and texture analysis. To my knowledge, the engineering background of the research content in the paper is very complex, and the development of cracks requires at least three stages: guided cracks, initial cracks, and crack expansion. However, the conclusions of this paper are lack of convincing and there are confusions in the paper that need to be clarified. The following are suggestions for revision:

Thank you very much for reviewing the manuscript and giving us the opportunity to revise and improve its quality. We have revised the article in terms of technology and writing. Please review it.

Issue 1. English grammar is quite chaotic, and English translation requires professional proofreading.

*Response*:

Due to language constraints, there are many grammatical problems in our paper, which makes it difficult to understand. We apologize for this. We carefully revised the whole article after checking for grammar errors, incorrect words, and other problems. We have invited the AJE organization to proofread our paper; specifically, this organization ensures that native speakers of English linguistics have revised and proofread the whole article. Modifying the readability and logic of the paper can arouse the interest of a wide range of readers, make the article better show our phased achievements, and provide a new research method for relevant researchers, as shown in Figure 1.

Figure 1. Proof of proofreading

Issue 2. During the coal rock blasting process, similarity ratio, materials, and model size have a significant impact on the research results. The development of cracks is a very complex process. It is questionable to what extent such a small model experiment can reflect the actual situation.

*Response*:

Thank you for your valuable advice. The materials used in tests were artificial coal-like materials. Coal is a heterogeneous brittle material and tends to lead to poor reproducibility of experiments [1]. The fracturing process requires extremely strict initial conditions, and even minor damage to coal rock can cause changes in the direction of cracks. The joint surface and original pore structure of coal can seriously affect the development of the strain field during the fracturing process. If we obtain different evolution laws under the same experimental conditions, then research is meaningless. Under the repeatability requirements of scientific experiments, we must simplify complex conditions. Only when homogeneous materials are studied clearly can we analyze heterogeneous materials.

In recent years, to reduce the mechanical strength and other important properties of coal seams as much as possible to the laboratory scale, scholars have conducted a large number of studies on the preparation of coal-like materials. For example: sand, lime, gypsum (Wang G., 2017) [2]; crushed coal, cement, activated carbon (Wang G., 2017) [3]; sand, paraffin (Wang X., 2017) [4]; cement, sand, coal powder, gypsum (Zhai C., 2018) [5].

We found some similar CO_2_ fracturing test equipment in the laboratory.

Table 1 Differences and verification with other experimental systems

| Authors | Specimen/material | Size/cm | System | The similarity in the results |
| --- | --- | --- | --- | --- |
| Ishida T, et al. [6] | granite | 17*17*17 |  | Small-size samples were easy to form through cracks, and the cracks were complex. |
| Ranjith P G, et al. [7] | coal-like materials | d=3.8  h=7.6 |  | With the CO_2_ pressure, the radial strain of coal increased, and the direction of the strain field inclined. |
| Kizaki A, et al. [8] | granite, tuff | 15*15*15 |  | CO_2_ fracturing was more likely to form fracture networks than hydraulic fracturing. |
| Zhou J, et al. [9] | shale | d=10  h=20 |  | CO_2_ fracturing was more effective, and more complex cracks were formed. |
| Zhang X, et al. [10] | shale | 20*20*20 |  | Increased injection pressure made material failure more obvious. The crack occurs at the weak position near the drill hole. |
| Wang L, el al. [11] | concrete | 20*20*20 |  | The fracture plane was rough, and the crack development was random, under low confining pressure and small stress difference. |

Issue 3. What is the connection between model testing and actual mines? How to consider the material and related rock mass structure of coal mines? Only using a single method to study precursor characteristics lacks innovation.

Thank you for your valuable opinions. The questions you raised are exactly what we want to study in depth in the future. In previous studies, our team developed a small-size test platform, carried out some tests of parameters such as vibration, displacement, crack development width and length [12-16]. The fracture initiation pressure model and gas pressure distribution model proposed in this paper have been well verified in laboratory experiments.

Our initial pressure was selected based on the similarity ratio ladder according to the actual situation of the field engineering test. There are some explanations as follows:

To explore the characteristics of fracturing pressure, initial pressures of 0.5 MPa, 1.0 MPa, and 1.5 MPa were selected to carry out the tests. Other scholars have also used these three typical pressures [13, 14]. Scholars have carried out experiments of CO_2_ blasting in the range of 1.2 MPa - 3.0 MPa. When the pressure is 1.2 MPa - 1.8 MPa, it is the crack initiation stage of the borehole fracture zone [17]. Therefore, we focused on the analysis of experimental data under 1.5MPa conditions.

Since the on-site situation is relatively complex, this paper first completes the derivation of theoretical formulas and laboratory experiments, and proposes a new method for monitoring coal and rock cracking initiation and crack propagation. In subsequent research, the model will be improved based on actual on-site conditions.

We have added explanations in **Discussion** section.

The limitations of this study are as follows:

(1) The effectiveness of gas extraction in coal seams is an important indicator for evaluating fracturing. In this study, the development of fractures, changes in mechanical parameters, and the morphology of damaged areas were mainly studied. The effectiveness of gas extraction should also be considered in the future.

(2) Coal is a heterogeneous material, and the joint surface and initial pore structure may affect the fracture failure area. There is insufficient research on heterogeneous materials fracture.

Given the limitations of the current research, we are currently improving the experimental equipment and carrying out some related work:

(1) Carry out large-scale physical similarity simulation experiment, by applying CH_4_ to the system before conducting the experiment to saturate the sample. CO_2_ and CH_4_ concentrations are measured before and after fracturing to evaluate the effectiveness.

(2) Samples using different materials are being made, and the development characteristics of cracks on heterogeneous interfaces are being studied by designing and adding gangue layers, faults, etc.

(3) By adding more non-destructive detection equipment, including but not limited to electronic probes, borehole peepers, non-destructive scanning equipment, etc. The development morphology of fractures can be obtained in real-time, and a 3-D simulated fracture network model can be established.

Thank you again for your selfless review of the manuscript and for providing references with high relevance and learning value. The review comments sincerely and carefully pointed out the main problems existing in the paper. By studying the references provided and carefully revising them, the overall level of the paper has been greatly improved. The continuous improvement of the paper makes our research more conducive to the reading of a wide range of readers and can inspire new ideas for relevant research.

***References***

[1]	Dawei Yin, Shaojie Chen, Yao Ge, et al. Mechanical properties of rock-coal bi-material samples with different lithologies under uniaxial loading[J]. Journal of materials research and technology, 2021, 10(1): 322-338.

[2]	Wang G, Wu M, Wang R, et al. Height of the mining-induced fractured zone above a coal face[J]. Engineering Geology, 2017, 216: 140-152.

[3]	Wang G, Li W, Wang P, et al. Deformation and gas flow characteristics of coal-like materials under triaxial stress conditions[J]. International Journal of Rock Mechanics and Mining Sciences, 2017, 91: 72-80.

[4]	Wang X, Liu X, Wang E, et al. Experimental research of the AE responses and fracture evolution characteristics for sand-paraffin similar material[J]. Construction and Building Materials, 2017, 132: 446-456.

[5]	Zhai C, Xu J, Liu S, et al. Fracturing mechanism of coal-like rock specimens under the effect of non-explosive expansion[J]. International Journal of Rock Mechanics and Mining Sciences, 2018, 103: 145-154.

[6]	Ishida T, Aoyagi K, Niwa T, et al. Acoustic emission monitoring of hydraulic fracturing laboratory experiment with supercritical and liquid CO_2_[J]. Geophysical Research Letters, 2012, 39(16).

[7]	Ranjith P G, Perera M S A. A new triaxial apparatus to study the mechanical and fluid flow aspects of carbon dioxide sequestration in geological formations[J]. Fuel, 2011, 90(8): 2751-2759.

[8]	Kizaki A, Tanaka H, Ohashi K, et al. Hydraulic fracturing in Inada granite and Ogino tuff with super critical carbon dioxide[C]//ISRM International Symposium-Asian Rock Mechanics Symposium. ISRM, 2012: ISRM-ARMS7-2012-109.

[9]	Zhou J, Liu G, Jiang Y, et al. Supercritical carbon dioxide fracturing in shale and the coupled effects on the permeability of fractured shale: An experimental study[J]. Journal of Natural Gas Science and Engineering, 2016, 36: 369-377.

[10]	Zhang X, Lu Y, Tang J, et al. Experimental study on fracture initiation and propagation in shale using supercritical carbon dioxide fracturing[J]. Fuel, 2017, 190: 370-378.

[11]	Wang L, Yao B, Xie H, et al. Experimental investigation of injection-induced fracturing during supercritical CO_2_ sequestration[J]. International Journal of Greenhouse Gas Control, 2017, 63: 107-117.

[12]	Wang K, Pan H, Zhang T, et al. Experimental study on the radial vibration characteristics of a coal briquette in each stage of its life cycle under the action of CO_2_ gas explosion[J]. Fuel, 2022, 320: 123922.

[13]	Wang K, Pan H, Zhang T. Experimental study of prefabricated crack propagation in coal briquettes under the action of a CO_2_ gas explosion[J]. ACS omega, 2021, 6(38): 24462-24472.

[14]	Pan H, Li J, Zhang T, et al. Study on crack propagation of the CO_2_ presplitting blasting empty hole effect in coal seam[J]. Energy Science & Engineering, 2020, 8(11): 3898-3908.

[15]	Li J, Pan H, Gong W, et al. Study on rock breaking characteristics of stress wave and blast-induced gas during CO_2_ gas blasting[J]. Journal of Petroleum Science and Engineering, 2022, 219: 111142.

[16]	Pan H, Qin B, Zhang T, et al. Influence of CO_2_ gas explosion on moulded-coal crack propagation under static load[J]. Journal of Xi'an University of Science andTechnology, 2022, 42(2),251-259. DOI:10.13800/j.cnki.xakjdxxb.2022.0208.

[17]	Wen H, Li Z B, Wang Z, et al. Experiment on the liquid CO_2_ fracturing process for increasing permeability and the characteristics of crack propagation in coal seam [J]. Journal of China Coal Society, 2016, 41(11):2793-2799. DOI: 10.13225/j.cnki.jccs.2016.0124.
