# Supplementary material for: Experimental study of precursory features of CO2 blasting-induced coal rock fracture based on grayscale and texture analysis
Source: PLoS One. 2024 Feb 9;19(2):e0297753. doi: 10.1371/journal.pone.0297753 (PMC10857609; doi:10.1371/journal.pone.0297753)
Supplement: Attachment — Submitted filename: Response to Reviewers.docx [file pone.0297753.s007.docx]

Manuscript Title: Experimental study of precursory features of CO_2_ blasting-induced coal rock fracture based on grayscale and texture analysis

Manuscript ID: PONE-D-23-21600

**Review comments**

Thank you for submitting your manuscript to PLOS ONE. After careful consideration, we feel that it has merit but does not fully meet PLOS ONE’s publication criteria as it currently stands. Therefore, we invite you to submit a revised version of the manuscript that addresses the points raised during the review process.

Thanks to authors for revising the article. It is further suggested to discuss and corelate this article with the SDG goals defined by UN and the concept of Circular Economy. Following studies are suggested for reference purpose and may be cited.

https://doi.org/10.1016/j.envres.2023.115523

https://doi.org/10.1007/s10570-023-05068-0

https://doi.org/10.1080/21655979.2023.2242124

https://doi.org/10.1016/B978-0-323-91187-0.00002-3

*Response*:

Thank you very much for reviewing the manuscript and giving us the opportunity to revise and improve its quality.

We have carefully reviewed the references on the Sustainable Development Goals and the concept of Circular Economy, gaining a clear understanding. Researchers have shown technological advancements in extracting valuable materials from various waste resources, providing important references for our study.

Our research aims not only to enhance coal seam permeability and gas extraction efficiency but also to promote sustainable development and circular economy practices. CO_2_ blasting, an eco-friendly technology, improves resource utilization and helps reduce greenhouse gas emissions. In line with the UN goals, our work highlights the potential of innovative energy technologies in environmental protection and efficient resource use.

We have revised the **Introduction** section of our article accordingly and invite you to review it.
